# Acknowledgment to Reviewers of *Antioxidants* in 2020

**DOI:** 10.3390/antiox10020170

**Published:** 2021-01-25

**Authors:** Antioxidants Editorial Office

**Affiliations:** MDPI AG, St. Alban-Anlage 66, 4052 Basel, Switzerland

Peer review is the driving force of journal development, and reviewers are gatekeepers who ensure that *Antioxidants* maintains its standards for the high quality of its published papers. Thanks to the cooperation of our reviewers, in 2020, the median time to first decision was 14 days and the median time to publication was 33 days. The editors would like to express their sincere gratitude to the following reviewers for their precious time and dedication, regardless of whether the papers were finally published:
Abdelmohsen, Usama RamadanLiverani, ElisabettaAbenavoli, LudovicoLivrea, Maria AntoniaAbraham, NaderLizard, GérardAbu-Reidah, Ibrahim M.Lloyd, MatthewAcedo, Jeella Z.Lo Muzio, LorenzoAčkar, ĐurđicaLobanova, Ekaterina S.Acquah, CalebLobo, GloriaAcuña-Castroviejo, DaríoLobo, JoaoAdamakis, Ioannis-DimosthenisLoboda, AgnieszkaAdamek, AgnieszkaLobysheva, IrinaAdamska-Patruno, EdytaLocato, VitoriaAdigun, OludoyinLodge, John K.Adornetto, AnnagraziaLodovici, MauraAfonso, Andrea Luísa FernandesLodyga-Chruscinska, ElżbietaAgnetti, GiulioLofrumento, Dario D.Agnieszka, SzopaLognay, GeorgesAichinger, GeorgLohan, SilkeAiello, FrancescaLoizzo, Monica R.Aimo, AlbertoLoizzo, Monica RosaAires, AlfredoLombardo, MauroAitken, R. JohnLomonaco, TommasoAlaimo, AlessandroLonardo, AmedeoAlamprese, CristinaLončar, Mirela BausAlbano, EmanueleLončarić, AnteAlbertini, MariaLongo, PasqualeAlbrecht, JanLongone, PatriziaAlcaraz, María JoséLopez Domenech, SandraAldasoro, MartinLópez Lluch, GuillermoAlexovič, MichalLopez Olvera, Jorge RamonAllegra, MarioLopez Rodilla, Jesus M.Allerton, TimothyLópez, Lucía MéndezAlloatti, GiuseppeLopez, VictorAlloza, IraideLopitz Otsoa, FernandoAllu, Prasanna K.R.Lorca, RamonAl-Maharik, NawafLordan, RonanAlmajano, María PilarLorenc-Koci, ElżbietaAl-Mrabeh, AhmadLorenzo, Jose M.Almstrup, KristianLu, Chi-YuAlonso-Alconada, DanielLu, Mei-ChinAlonso-Moreno, CarlosLubrano, ValterAlonso-Torre, SaraLuca, GiovanniAlquezar-Burillo, CarolinaLucarini, MassimoAltamimi, M.Lucero, DiegoAlvarez Fernandez, María AntoniaŁuczaj, WojciechAlvarez-Suarez, JoseLuddi, AliceAlves, C. HenriqueLudwig, BerndAlves-Lopes, RheureLuís, ÂngeloAlves-Silva, Jorge MLukasiewicz, MarcinAmaral, AlexandraLukaski, HenryAmaral, Joana S.Lukyanov, Pavel A.Amarowicz, RyszardLuna Maldonado, AurelioAmillis, SotirisLund, Elizabeth K.Amiya, EisukeLung, Ming-YuAmmendola, RosarioLunov, OlegAmoaku, Winfried M.K.Lupi, Saturnino MarcoAmodio, NicolaLupiáñez, José A.Amorati, RiccardoLuzina, OlgaAmorós, AsunciónLymberopoulos, AristotelisAmpofo, EmmanuelLynch, Sean M.Anandhan, AnnaduraiM. Vorob’ev, MikhailAncuceanu, RobertMa, SihuiAndrade, IsabelMaccallini, CristinaAndre, FilipaMacchi, BeatriceAndrei, SandaMachado Araújo, Maria EduardaAndreou, EleniMachin, Daniel R.Angelino, DonatoMaciejczyk, MateuszAngeloni, GiuliaMackrill, JohnAngius, FabrizioMadej, DawidAnifandis, GeorgeMademtzoglou, DespoinaAnisimova, IrinaMadigan, MicheleAnnibalini, GiosuèMadrid, MarisaAnnunziata, GiuseppeMadureira, PatriciaAnsari, RaisMaeng, Sung HoAntal, DianaMaffei, GianlucaAntipova, VeronicaMaffia, MicheleAntognelli, CinziaMagierowski, MarcinAntolak, HubertMagni, PaoloAnton, PaulineMaher, PamelaAntonelli Incalzi, RaffaeleMahy, Jean-PierreAntonini, GiovanniMaietti, AnnalisaAppell, MichaelMailloux, RyanAprile, Francesco A.Maio, Roberto DiAprodu, IulianaMaioli, MargheritaAratani, YasuakiMaioli, SilviaAraujo, Maria E.M.Maione, FrancescoArcone, RosariaMaiuolo, JessicaArellano, Juan B.Maixent, Jean MichelArese, MarziaMajima, Hideyuki J.Arguelles, SandroMajolino, DomenicoArguello, JoseMakarova, KaterinaArion, FelixMakedou, KaliArita, MasanoriMakishima, MakotoArmand, Anne-SophieMalapelle, UmbertoArmand, MartineMalinska, HanaArmeni, TatianaManam, SrikanthArmindo Melo, ArmindoMancias, Joseph D.Arndt, Patrick G.Mancini, AntonioArner, EliasMancini, InesArnhold, JuergenMandal, AmritlalArsene, Andreea LetiţiaMandatori, DomitillaArtín-Aragón, Sagrario M.Mandrich, LuigiAssari, ShervinMandrioli, RobertoAston, Kenneth I.Máñez, SalvadorAsuero, Agustín G.Manfredini, StefanoAtalay, MustafaManini, PaolaAthanasiadis, VassilisManjunath, ManuboluAthanasiou, Labrini V.Mann, FrancisAtlante, AnnaMann, GiovanniAudrito, ValentinaManna, CaterinaAugé, NathalieMannino, GiuseppeAydillo, CarlosMantell, CasimiroAykin-burns, NukhetMantzouridou, FaniAzab, WalidMarabini, LauraAzadi, HosseinMaranesi, MargheritaAzzimato, ValerioMarasco, DanielaAzzini, ElenaMarcal, HelderBabickova, JankaMarchetti, NicolaBacchetti, TizianaMarchini, CristinaBacci, StefanoMarco-Contelles, JoséBach, Duc-HiepMarek, KieliszekBąchor, RemigiuszMarengo, BarbaraBácskay, IldikóMaresca, MarcBadea, MihaelaMarfany, GemmaBadino, PaolaMargaritelis, NikosBae, Dong-HunMarginǎ, DenisaBae, Hae-RahnMari, MicheleBae, JeehyeonMaria Carmela, Bonaccorsi Di PattiBae, Ok-NamMaría Rosaura, Leis TrabazoBagci, SoyhanMarini, FedericoBagdziunas, GintautasMarini, Herbert RyanBagnicka, EmiliaMarino, JosephBagnoli, PaolaMarino, TizianaBai, RenrenMariot, VirginieBailey, Charles GMariscal-Arcas, MiguelBajek, AnnaMarkopoulos, Georgios S.Bajinskis, AinarsMarletta, LuisaBajor, MalgorzataMarra, AlbertoBaki, GabriellaMarracci, SilviaBalan, PrabhuMarrano, NicolaBalasubramanian, BalamuralikrishnanMarsili, StefaniaBalcerczyk, AnetaMarsillach, JuditBaldanzi, GianlucaMarsol, AlexisBaldisserotto, AnnaMarszalek, MartaBálint, ErikaMartelletti, PaoloBališ, PeterMartí, SergioBalzano, TizianoMartikainen, RiikkaBambling, MatthewMartin, EricBandyopadhyay, DebasishMartin, MariaBanerjee, AditiMartin-Bastida, AntonioBanti, Christina N.Martínez-Espinosa, Rosa MaríaBarba, Francisco J.Martínez-González, JoséBarbosa-Pereira, LetriciaMartinez-Hervas, SergioBarbouti, AlexandraMartinez-Martos, Jose ManuelBarca, AmilcareMartinez-Monteagudo, Sergio I.Bárcena, José AntonioMartinez-Pastor, FelipeBarker, TylerMartínez-Sánchez, GregorioBarragán, MontserratMartín-Gil, JesusBarreca, DavideMartini, DanielaBarreto, Maria CarmoMartins, Alice M.Barritt, GregMartins, ArturBartella, LuciaMartins, IanBartelt, AlexanderMartins, NatáliaBartolini, SusannaMarty, GaryBartosz, GrzegorzMarullo, SalvatoreBartoszewski, RafalMaruyama, KeiBaschieri, AndreaMasłyk, MaciejBasile, AdrianaMast, ThomasBasile, TeodoraMastinu, AndreaBaskaran, RathinasamyMasurier, NicolasBasu, ReetobrataMateeva, NellyBatchelor, PaulMateos Bernal, Rosa MariaBatinic-Haberle, InesMaterska, MałgorzataBattafarano, GiuliaMateus, Maria L.Battistuzzi, GiannantonioMateus, NunoBauer, GeorgMathew, Anna V.Bauer, IngeMathew, MonaBauer, MarioMatilla, MiguelBax, Bridget E.Matkowski, AdamBaydur, AhmetMatraszek-Gawron, RenataBazwinsky-Wutschke, IvonneMatsuda, AtsushiBeberok, ArturMatsugo, SeiichiBecatti, MatteoMatsumoto, YoshinoriBednar, PetrMatsunami, KatsuyoshiBedon, FrankMatsuo, MuneakiBeharry, Kay D.Matsuzaki, ShinyaBehringer, ErikMattii, LetiziaBeis, DimitrisMattioli, Anna VittoriaBellezza, IlariaMattoscio, DomenicoBellier, Jean PierreMatuszewska, AnnaBellosta, PaolaMaugeri, GraziaBelyaeva, Olga V.Maupin-Furlow, Julie A.Bendich, AdrianneMauriz, José L.Benedec, DanielaMauro, Adolfo GabrieleBenedetti, FrancescaMaxwell, Jessie R.Benhar, MoranMayer-Proschel, MargotBenito, José ManuelMayhan, William G.Bennett, ALAN B.Mayo, Juan CarlosBenohoud, MeryemMazur-Bialy, AgnieszkaBenoist, HervéMcAllister, MattBenson, DavidMcDonald, DeniseBentel, Jacqueline M.McGill, Mitchell R.Berardesca, EnzoMcMillan, JoEllyn M.Berardo, ClarissaMcmorrow, TaraBergantini, LauraMd Badrul, AlamBerger, Daniela CristinaMeaney, SteveBerger, TrishMears, JasonBeriáin Apesteguía, María JoséMeccariello, RosariaBermudez, MarcelMedina, Reinhold J.Bernabò, NicolaMehariya, SanjeetBernardini, GiuliaMehta, SunaliBernátová, IvetaMeléndez-Hevia, EnriqueBernocchi, GraziellaMelini, ValentinaBernstein, NiritMeloni, BrunoBerreau, Lisa M.Mena, SalvadorBerti, FedericoMencke, RikBertocchi, MartinaMéndez, AnaBezirtzoglou, EugeniaMeneguzzo, FrancescoBharath, Leena P.Menendez, Jose CarlosBhattacharyya, PiyaliMenghini, LuigiBhatti, Fazal-ur-RehmanMennerich, DanielaBiagini, GiuseppeMerah, OthmaneBiałek, AgnieszkaMercolini, LauraBialonska, DobroslawaMerecz-Sadowska, AnnaBianco, ArmandodorianoMerry, TroyBidault, GuillaumeMertas, AnnaBielas, WiesławMesalam, AhmedBielle, FranckMeslet-Cladiere, LaurenceBijak, MichałMesnage, RobinBikov, AndrásMessner, BarbaraBilski, JanMessner, DonaldBink, Diewertje IMetere, AlessioBird, AmandaMetzinger, LaurentBirringer, MarcMeurman, JukkaBirsa, Mihail LucianMeynier, AnneBisti, SilviaMézes, MiklósBiswas, ArijitMiastkowska, MałgorzataBito, TomohiroMicale, NicolaBjerregaard, PoulMicek, AgnieszkaBjornstedt, MikaelMiceli, NataliziaBlack, Stephen M.Michael, AdrianBlackstone, CraigMichaelis, MartinBlais, AnneMichalopoulos, IoannisBlalock, WilliamMichalska, AnnaBlando, FedericaMichaud, PhilippeBlangetti, MarcoMichel, PiotrBlank, RalfMicheli, LauraBlanton, CynthiaMichels, Alexander J.Blasi, FrancescaMicillo, RaffaellaBlažević, IvicaMicucci, MatteoBleilevens, ChristianMielcarek, MichalBloksgaard, MariaMieyal, John J.Bloor, StephenMigliori, Carmela AnnaBoard, MaryMiguel, Maria Da Graça CostaBoarescu, Paul-MihaiMíguez, Jesús M.Boaz, MonaMikelis, ConstantinosBobis, OtiliaMikstacka, RenataBochkov, ValeryMikulic-Petkovsek, MajaBocian, SzymonMilano, SerenaBoczkowska, MajaMilardi, DaniloBoffa, LuisaMilković, LidijaBogdański, PawełMillar, J. CameronBöhm, VolkerMiloso, MariarosariaBohn, TorstenMimura, JunseiBoltze, JohannesMin, HyeyoungBombardi, CristianoMinami, MasaakiBömer, NilsMinato, Ken-ichiroBoni, RaffaeleMinetti, GiampaoloBonini, Sara AnnaMiniero, Daniela ValeriaBonior, JoannaMinnelli, CristinaBonnländer, BerndMioc, MariusBonsembiante, FedericoMiquel, AdroverBoo, Yong ChoolMiranda, JonatanBora, Puran S.Miranda, MaríaBorbély, YvesMironeasa, SilviaBordes, María Dolores LlobatMirza, Muhammad UsmanBordin, LucianaMisawa, ErikoBordonaro, MichaelMishima, EikanBorghini, AndreaMishra, BirendraBorkum, Jonathan M.Mishra, RosalinBorras, ConsueloMitani, TakakazuBorrelli, EmmaMitchell, Scott G.Bosca, LisardoMitliagka, ParaskeviBoschi-Muller, SandrineMiyamoto, KojiBotez, ElisabetaMiyazawa, ShinichiBottani, EmanuelaMiziak, BarbaraBottone, Maria GraziaMizobata, TomohiroBouet, ValentineMladěnka, PřemyslBourdon, EmmanuelMobbili, GiovannaBouropoulos, NikolaosMoccia, MarcelloBoyden, Penelope A.Modesti, AlessandraBozic, JoskoModica, MariaBracco, EnricoModun, DarkoBradshaw, NicholasMogoșan, CristinaBradshaw, PatrickMohamed, TamerBrady-Kalnay, Susann M.Mohanty, Joy G.Braidy, NadyMoise, AlexanderBraig, SimoneMoldovan, BiancaBrainina, Khiena Z.Moldoveanu, Serban C.Branco, VascoMolinaro, AntonioBrandli, AliceMolins, BlancaBrandt, KirstenMollace, VincenzoBrash, AlanMollen, Kevin P.Brasili, ElisaMollica, AdrianoBratek-Skicki, AnnaMolmenti, Ernesto P.Bravo, JeronimoMolnar, ArpadBravo, Susana B.Mołoń, MateuszBrčić Karačonji, IrenaMomand, JamilBrebu, MihaiMoncharmont, BrunoBreitbart, HaimMondal, KunalBrestic, MarianMondanelli, GiadaBrett, ThomasMontana, GiovannaBromfield, ElizabethMontemiglio, LindaBroskey, Nicholas T.Montenegro, LuciaBrown, DennisMontesano, DomenicoBrown, Kyle E.Montes-García, VerónicaBrown, LaVerne L.Monticone, SilviaBrownlee, Iain A.Montiel-Duarte, CristinaBrukman, NicolasMontone, Carmela MariaBrullo, ChiaraMonzón, MartaBrunetti, CeciliaMookerjee, ShonaBrunetti, GiacominaMoon, Jong-SeokBrunetti, Luigi (Italy)Moon, MinhoBrunetti, Luigi (USA)Moore, Roger A.Bruno, LauraMoore, Tiffany A.Bruns, Danielle R.Morabito, RossanaBrycki, BogumilMorales Suárez-Varela, María M.Bu, PengliMorbidelli, LuciaBucekova, MarcelaMorciano, GiampaoloBucevičius, JonasMoreno, Diego A.Buchet, ReneMoreno, J.J.Buchner, PeterMoreno, Maria JoãoBuck, LeslieMoreno, SandraBucolo, ClaudioMorgan-Bathke, MariaBudzisz, ElżbietaMorikawa, SatoruBueno Dalto, DanyelMorimura, ShigeruBuentzel, JensMorrell, Jane M.Buey, Rubén M.Morrow, John P.Buga, AnaMoschini, RobertaBuga, Ana-MariaMosgöller, WilhelmBullon, PedroMoshage, HanBülow, Margret H.Mosharov, Eugene V.Bunea, AndreaMoskalev, Alexey A.Bunik, VictoriaMoskovitz, JackobBurcul, FrankoMosqueira, MatiasBurns, DavidMot, AugustinBurns, Teresa A.Motterlini, RobertoBusco, GiovanniMougios, VassilisBusinaro, RitaMousa, Shaaban A.Butcher, AdrianMrakic-Sposta, SimonaButler, Thomas O.Mravljak, JanezButterfield, David AllanMshvildadze, VakhtangCaballero-Garcia, BeatrizMuceniece, RutaCabeza, LauraMucha, JoannaCabiscol, ElisaMücke, RalphCaccamo, DanielaMueller, Wolf C.Cacciola, FrancescoMugisho, OdunayoCachofeiro, Victoria M.Mu-Hsin, ChangCádiz-Gurrea, María De La LuzMulas, MaurizioCagliero, Cecilia LuciaMulinacci, NadiaCahill, PaulMuller, Manfred JamesCai, WenlongMuller, Patricia A. J.Caiati, CarloMüller-Schüssele, StefanieCaiazzo, ElisabettaMuñiz, PilarCaires, Hugo R.Muntean, DaninaCairo, GaetanoMunteanu, Florentina-DanielaCairrão, ElisaMura, Maria ConsueloCalabrese, Edward J.Mura, UmbertoCalcabrini, CinziaMurakami, KoheiCaldara, MarinaMurakami, YasufumiCaliceti, CristianaMurasami, AkiraCalina, DanielaMurase, SachikoCalvo-Lobo, CésarMurata, KazuyaCametti, CesareMurawska-Ciałowicz, EugeniaCamins, AntoniMurdaca, GiuseppeCammisotto, VittoriaMurdica, ValentinaCampbell, Fiona M.Murphy, RonanCampos Rosa, Joaquín MaríaMuscella, AntonellaCampos, DeboraMusco, NadiaCamps, JordiMustachio, LisaCañadas, OlgaMustonen, Anne-MariCancello, RaffaellaMuszyńska, EwaCangkrama, MichaelMuszyński, SiemowitCanini, AntonellaMutus, BulentCannavò, Serafinella P.Muzio, GiulianaCantini, FrancescaMuzolf-Panek, MalgorzataCao, JianMyasoedova, VeronikaCapasso, RaffaeleMysliveček, JaromírCapó, XavierNabika, ToruCarbone, KatyaNabissi, MassimoCardenia, VladimiroNagahara, NoriyukiCardoso, Isabel SantosNagai, TakeharuCardoso, SusanaNagata, KoseiCardullo, NunzioNagy, BálintCarelli, StephanaNagy, Előd ErnőCarles Escola-Gil, JoanNagy, IstvanCarlisi, DanielaNajera, FranciscoCarlotto, SilviaNakajima, IkuyoCarluccio, Maria AnnunziataNakaki, ToshioCarmeli, ShmuelNakamura, MotokiCarmen, MejíaNakamura, SoichiroCarnevale Neto, FaustoNakata, MasanoriCarnevale, RobertoNakatani, YoshihikoCaroppo, EttoreNakatsu, KanjiCarotenuto, MarcoNalivaeva, NataliaCarotti, SimoneNamisaki, TadashiCarradori, SimoneNandi, ShyamCarrera Fernández, CeferinoNannoni, EleonoraCarrera, MónicaNapolitano, AlessandraCarresi, CristinaNapolitano, GaetanaCarrillo, CeliaNarayan, MaheshCarrizzo, AlbinoNarayanan, S. PriyaCarstensen, Hans HeinrichNardecchia, StefaniaCarullo, GabrieleNasim, Muhammad JawadCaruso, GiuliaNatarajan, SivaramanCarvalho, Andreia N.Nath, ArijitCarvalho, DanielNaumovski, NenadCarvelli, LuciaNavarro-Alarcon, MiguelCasalino, ElisabettaNavas, PlacidoCasamassimi, AmeliaNaviglio, DanieleCasanova, LlanosNawirska-Olszańska, AgnieszkaCasanova-Sáez, RubénNeag, Maria AdrianaCasas, Rosa M.Neale, Joseph H.Caseiro, ArmandoNegishi, TakayukiCasella, LuigiNegro, CarmineCaser, MatteoNemoto, KiyomitsuCasey, JenniferNenadis, NiknenCaspari, ThomasNeuditschko, MarkusCastellano, ImmacolataNeuman, ManuelaCastillo-Gonzalez, Claudia MarcelaNeumann, JakeCastñeiras, AlfonsoNg, Hooi HooiCatarino, Marcelo D.Nicholas, DarrelCatucci, GianlucaNicoletti, FerdinandoCaulfield, Thomas R.Niculescu, LoredanCauli, OmarNiederberger, EllenCavaliere, GinaNieto, GemaCavallaro, SebastianoNieto, Marcelo J.Cavoski, IvanaNieva-Echevarría, BárbaraCejkova, JitkaNikkanen, LauriCeranowicz, PiotrNikolakopoulou, Angeliki M.Cerqueira, FatimaNioi, ClaudiaCervantes, EmilioNishi, KosukeCervellati, CarloNishi, MayumiCervelli, ManuelaNishijima, KazutoshiCervera, AnaNishikawa, YoshikazuCesa, StefaniaNishimura, KojiCesaro, AnnabelleNishimura, NaomichiChae, HeeyoungNissen, LorenzoCháfer-Pericás, ConsueloNiu, TianhuaChaki, MouniraNiwano, YoshimiChami, BelalNjus, DavidChan, CatherineNoain, DanielaChand, Kirat K.Nobile, StefanoChang, Cheng-ChungNocella, CristinaChang, Chia-cheNoda, TakeshiChang, Geng-RueiNofer, Jerzy-RochChang, Hsin-I.Noguchi, Constance TomChang, Hsueh-WeiNoguera Artiaga, LuisChang, Jer-MingNogues, IsabelChang, Kee-lungNoh, Ji-yoonChang, Kun-CheNoren Hooten, NicoleChang, Ling-ChuNoutsios, GeorgiosChang, Te-ShengNouvian, RegisChang, Yuan-YenNoviello, CarmineChano, TokuhiroNovikov, Arthur I.Chao, Louis KuopingNowacka-Jechalke, NataliaChapple, SarahNowak, DorotaChartoumpekis, DionysiosNowak, RenataChatzinikolaou, AthanasiosNowakowska-Zajdel, EwaChatzopoulou, PaschalinaNowicka-Bauer, KarolinaChavatte, LaurentNowomiejska, KatarzynaChekanov, KonstantinNucera, EleonoraChen, Chiung-MeiNunez, OscarChen, Chung-HwanNunzio, VicarioChen, Chun-JungNuzzo, DomenicoChen, DavidNwaiwu, OgueriChen, GuibingNycz, Jacek EChen, Hsiao-Jou CortinaNylander, KarinChen, Hsing-YinO’Keeffe-Ahern, JonathanChen, Jen-TsungO’Leary, SeánChen, Jing-HsienO’Shaughnessy, RyanChen, Liang-YuObana, AkiraChen, Shuen-EiOberemok, Volodymyr V.Chen, Wei-JuneOberley, RebeccaChen, XiaoyanObrador, ElenaChen, YihungOchi, ShinichiroChen, Yih-YuanOchoa, JulioChénais, BenoitOczkowski, MichałCheng, Juei-TangOda, ShojiCheng, Pei-WenOddo, ElisabettaCheng, Yuan-BinOgai, KazuhiroCheon, Yong-PilOgi, TakayukiCherkaoui Malki, MustaphaOgra, YasumitsuChhabra, GaganOh, Jae-WookChiang, Hsiu-MeiOhnishi, ShihoChiang, Wen-DeeOka, ShinichiChiaramello, AnneOkada, HiroshiChimienti, Guglielmina AlessandraOkajima, HideakiChinnici, FabioOksenych, ValentynChirikova, Nadezhda K.Okumura, NobuakiChistyakov, Vladimir AnatolievichOlaru, Octavian TudorelChitgupi, UpendraOlech, MartaChiu, Yi-HanOlennikov, DaniilChizzola, RemigiusOliveira, M. ConceiçãoChmielarz, PiotrOliver, Peter L.Cho, Jae YoulOmar, Syed HarisCho, JongkiOndrias, KarolCho, Somi KimOng, Eng ShiChobot, VladimírOniga, IlioaraChoi, JonghoonOniszczuk, AnnaChoi, Jong-ilOpaliński, ŁukaszChoi, Yoon KyungOppedisano, FrancescaChoi, Young HaeOracz, JoannaChoi, Yun SangOrfila, CarolineChong, Cher-RinOrlacchio, AntonioChou, Wen-HaiOrlando, GiustinoChrzanowski, GrzegorzOroian, Mircea AdrianChua, Horng RueyOrso, EvelynChung, Hun TaegOrsolic, NadaChung, Hwan-SuckOrtega, Angel L.Chyau, Charng-CherngOrtega, María JesúsCiaccio, MarcelloOrtega-Villaizan, Maria Del MarCianciolo, GiuseppeOrzelska-Górka, JolantaCiarcia, RobertoOsella, Alberto RubénCicchella, AntonioOsredkar, JoškoCicco, Paola DeOstacolo, CarmineCiccodicola, AlfredoOsuchowski, MarcinCiccone, MarcoOthman, Eman M.Çiçek, Serhat SezaiOtręba, MichałCicero, ArrigoOtsuka, YuzuruCichorek, MiroslawaOtulak-Kozieł, KatarzynaCichowska, JoannaPacak, ChristinaCicione, ClaudiaPace, PaulCiesla, LukasPacifico, SeverinaCilla, AntonioPacini, AlessandraCimino, FrancescoPadilla-Benavides, TeresitaCimmino, GiovanniPagano, GiovanniCimpoiu, ClaudiaPage, MelissaCioanca, OanaPagliarani, AlessandraCiobica, AlinPagonopoulou, OlgaCione, ErikaPak, Jhang HoCiornea, Elena TodirascuPalacios-Santander, José MaríaČipak Gašparović, AnaPalade, Laurentiu MihaiCipak, LubosPalestini, PaolaCipolloni, LuigiPallardó, Federico V.Ciz, MilanPallàs Lliberia, MercèCladis, DennisPallottini, ValentinaClanchy, FelixPalma, MiguelClemens, MarkPalmerini, MariaClifford, TomPalmieri, ErikaCocco, RaffaellaPaltinean, RamonaCocco, TizianaPalumbo, Giuseppe AlbertoCodella, RobertoPanagiotidis, Mihalis I.Codina, GeorgianaPanaro, Maria AntoniettaCohen, GuyPanfoli, IsabellaCole, MarshaPang, Cheng-YoongColell, AnnaPang, Myung-GeolColla, EmanuelaPannella, GianfrancoCollier, JasonPansarasa, OriettaColnaghi, LucaPantaleo, AntonellaColonna, Maria AntoniettaPantea Stoian, AncaColucci, GiuseppePanzella, LuciaComi, CristoforoPaola, Rosanna DiComincini, SergioPaoletti, Anna MariaComino, PennyPaoli, PaoloComizzoli, PierrePaolino, DonatellaConstantinescu-Aruxandei, DianaPapież, MonikaConstantinou, ViolettaPapoutsis, KonstantinosConsumi, MarcoPappa, AglaiaContardi, MarcoPappas, ChristosConte, IvanParađiković, NadaContino, MarialessandraParameshwaran, KodeeswaranContreras, María Del MarParenti, AstridCoombs, MelaniePariente Llanos, Jose AntonioCopeland, PaulPark, Chan YoungCopolovici, Dana MariaPark, Han-ACopolovici, LucianPark, Joon YoungCoppo, LuciaPark, Jun HyoungCoquerel, DavidPark, JunsooCorazzari, MarcoPark, Kwang-hyunCordani, NicolettaPark, Ky YoungCordaro, MarikaPark, Kyoung-ChanCorpe, ChristopherPark, Kyung-soonCorreia, Carlos M.Park, Sang-WookCorsi, LorenzoPark, Shin-HyungCorso, GiovanniPark, SongyoungCossignani, LinaPark, WansuCosta, RaquelParolini, CinziaCosta, RobertoParry, Traci L.Costantini, LaraParsonage, DerekCotârleț, MihaelaPârvu, Alina ElenaCottrell, JeremyPârvu, MarcelCouch, James R.Pasciu, ValeriaCoumans, Joëlle V. F.Pasman, Wilrike J.Couture, ManonPasqualone, AntonellaCouturier, JeremyPastore, AnalisaCozar, IPastorelli, GraziaCraig, MorriePateiro, MirianCraveiro, RitaPatel, AlokCravero, Maria CarlaPateras, Ioannis S.Cravotto, GiancarloPatruno, AntoniaCrespo, IrenePatruno, CataldoCrestani, MaurizioPattappa, GirishCrinelli, RitaPatyra, EwelinaCrispi, StefaniaPaudel, BishalCroft, Kevin D.Pauly, DianaCross, NeilPautz, AndreaCrupi, PasqualePaventi, GianlucaCrupi, RosaliaPavic, ValentinaCruz-Chamorro, IvanPavon-Djavid, GracielaCsányi, GáborPawlak, DariuszCuchillo, InmaculadaPawlas, NataliaCui, YePawliczak, RafalCullen, JosephPazdro, RobertCuomo, FrancescaPazzagli, LuigiaĆurko, NatkaPeana, Massimiliano F.Currò, MonicaPecorelli, AlessandraCurti, DanielaPeiretti, Pier GiorgioCurtis, Michael J.Peixoto, FranciscoCutler, Mary LouPék, ZoltánD. Bortner, CarlPelle, Flavio DellaD’Amico, StefanoPellegrini, MarikaD’Angelo, LiviaPellegrino, DanielaD’Angelo, RosaliaPellegrino, Roberto MariaD’Antona, NicolaPeluso, IlariaD’auria, EnzaPence, BrandtD’Emmanuele Di Villabianca, RobertaPenna, ClaudiaD’Orazi, GabriellaPenning, LouisD’Orazio, GiovanniPepe, SalvatoreD’Orsi, BeatricePerego, PaolaDa Silva, Luís PintoPereira, DavidDa Silva, Sabrina DanielaPereira-Leite, CatarinaDacheux, Jean-LouisPerera, Chamini J.Dagnell, MarkusPerera, ConradDal Monte, MassimoPerestrelo, RosaDalessandri, DomenicoPerestrelo, Rosa Maria De SáDalessandro, GiuseppePérez, SalvadorDall’Acqua, StefanoPérez-Alvarez, Jose AngelDall’Aglio, CeciliaPérez-Gálvez, AntonioDalla Pozza, ElisaPergolizzi, SimonaDamiano, FabrizioPerkins, AndrewDansette, Patrick M.Perkovic, IvanaDar, Wasim A.Permyakov, EugeneDario, BrunettiPero, RaffaelaDarmochwał-Kolarz, DorotaPerrotta, CristianaDarvesh, AltafPersichilli, SilviaDaugelavicius, RimantasPeskin, AlexanderDave, SandeepPetcherski, AntonDavies, MichaelPetit, Patrice X.Davis, Keith R.Petropoulos, IsabelleDavis, Randall L.Petropoulos, Spyridon ADawra, RajinderPetrosino, MariaDay, Andrew S.Petrotos, KonstantinosDe Bellis, LuigiPetrucci, RitaDe Falco, BrunaPetruczynik, AnnaDe Filippis, BarbaraPezeshki, AdelDe Geest, BartPezzuto, AldoDe Haan, JudyPhilips, NeenaDe Leonardis, AntonellaPiątkowska, EwaDe Lucca Camargo, LiviaPiccinelli, Anna LisaDe Martin, SaraPiccoli, MarcoDe Masi, LuigiPiekoszewski, WojciechDe Melo, Marcelo M.r.Piemontese, LucaDe Tullio, Mario C.Pierdomenico, Johnny DiDe Vendittis, EmmanuelePietrobelli, AngeloDe Waard, VivianPietrzak, WioletaDe-Bandt, Jean-PascalPignatti, PatriziaDeckert, JoannaPignitter, MarcDefant, AndreaPikija, SlavenDegan, PaoloPikuleva, IrinaDegl’innocenti, DonatellaPilipenko, VladimirsDekker, MarloesPilmane, MāraDel Castillo-Santaella, TeresaPiluzza, GiannellaDelcourt, NicolasPimienta, Rodney LacretDelis, CostasPina, MariaDell’Agli, MarioPinar, AnitaDelprat, BenjaminPinchuk, IlyaDelvin, EdgardPineda, MiguelDe-Miguel, ManuelPiñeiro, MartaDent, MajaPinela, JoséDerry, PaulPinheiro, JoaquinaDevesa, JesúsPini, Luigi AlbertoDevkota, Hari PrasadPintea, AdelaDevkota, PratimaPintus, ElianaDeWald, TracyPintus, GianfrancoDi Cerbo, AlessandroPinzaru, IuliaDi Giacomo, SilviaPiomboni, PaolaDi Gioia, Maria LuisaPiotrowska, KatarzynaDi Marco, StefanoPiotrowska, MałgorzataDi Maro, AntimoPirkmajer, SergejDi Martino, AntonioPirman, TatjanaDi Mattia, Carla DanielaPirozzi, ClaudioDi Natale, ConcettaPiscopo, MarinaDi Nunzio, MattiaPittalà, ValeriaDi Paola, RosannaPiu, SahaDi Pietro, NataliaPizent, AlicaDi Salle, AnnaPlastina, PierluigiDi Sotto, AntonellaPlatania, ChiaraDiaconeasa, ZoritaPlech, TomaszDiakopoulos, NinaPloner, ChristianDiaz-Vivancos, PedroPlumb, JennyDickerhof, NinaPluta, RyszardDidion, Sean P.Pluth, Michael D.Diederich, SandraPodgórska, AnnaDiego, La MendolaPodsiadły, RadosławDifonzo, GrazianaPoeggeler, BurkhardDikalov, SergeyPohl, PawelDimauro, IvanPohl, SebastianDinh, Quynh NhuPoiana, MarcoDini, IrenePoklar Ulrih, NatašaDinica, Rodica-MihaelaPolacek, NorbertDinis, Lia Tânia JPoli, GuiseppeDinis, MárioPolicar, ClotildeDintcheva, Nadka TzankovaPöling, JochenDireito, RosaPolonini, Hudson CaetanoDiSilvestro, RobertPolyakov, NikolayDobrikova, AneliaPoma, PaolaDodds, W. JeanPompella, AlfonsoDogossy, GáborPompili, ElenaDolashka, PavlinaPompili, SimonaDołowy, MałgorzataPons, ToniDomenicotti, CinziaPoole, Leslie B.Domínguez Medina, EduardoPop, AncaDominguez, Juan CarlosPop, CristinaDomínguez-Álvarez, EnriquePopa, Daniela-SavetaDomínguez-Perles, RaúlPopescu, SilvanaDomoki, FerencPorro, ChiaraDonati Zeppa, SabrinaPorteu, FrançoiseDonato, LuigiPósa, AnikóDonato, PaolaPosadino, Anna MariaDonato, RosarioPotaczek, Daniel P.Donato, Rosario FrancescoPotočnjak, IvaDonnini, SandraPourié, GrégoryDonno, DarioPovey, AndrewDos Santos, Jorge LuizPozsgai, GaborDosio, FrancoPrado, Armando Perez DeDOSOKY, NOURAPrado-Cabrero, AlfonsoDrabińska, NataliaPradotto, Luca GuglielmoDrenjančević, InesPrassl, RuthDrevinskas, TomasPrats, María SoledadDrosten, MatthiasPravenec, MichalDrozd, RadoslawPrieto, Miguel ÁngelDrużyńska, BeataPrieto, Rafael MariaDuarte, AmilcarPrimack, WilliamDuarte, DelfimPrimavera, RositaDubrova, YuriProcopio, Jesús R.Duda-Chodak, AleksandraProestos, CharalamposDudas, JozsefProietti, SilviaDudley, Samuel C.Przybył, JarosławDuer, StanisławPuglisi, IvanaDufossé, LaurentPula, GiordanoDuggan, Brendan MPulido, Olga MDugue, BenoitPulina, GiuseppeDuguez, StephaniePutilov, ArcadyDulf, FranciscPyka, AlinaDumitru, GabrielaPyka-Pająk, AlinaDuncan, Raymond ScottQiu, BinDuplessis, MélissaQuaglino, DanielaDurante, MirianaQuan, TaihaoDurante, WilliamQuartu, MarinaDuranti, GuglielmoQuax, PaulDuszka, KalinaQuiles Morales, Jose L.Dutartre, PatrickQuinlan, Leo R.Dutu, LigiaQuinn, KylieDuvigneau, J. CatharinaQuinto, Emiliano JoséDziadas, Mariusz TomaszQuiros-Gonzalez, IsabelDziekońska, AnnaR. El-Seedi, HeshamDziki, DariuszRacay, PeterDżugan, MałgorzataRaccuia, Salvatore AntoninoEckhart, LeopoldRachek, LyudmilaEdge, RuthRadak, ZsoltEfthimia, CotouRadko, LidiaEgawa, TatsuroRadulescu, CristianaEgerszegi, IstvánRaffaelli, NadiaEidenberger, ThomasRafińska, KatarzynaEilertsen, Karl-ErikRagino, Yuliya I.El Ghoch, MarwanRagusa, AndreaEl Midaoui, AdilRagusa, Maria AntoniettaElango, JeevithanRahimi, FaridElgendy, MohamedRajala, Raju V.S.Elisabetta, BraviRajauria, GauravElshabrawy, HatemRajput, Vishnu D.Elsner, MatthiasRalph, Stephen JohnEmri, EszterRamaiahgari, Sreenivasa C.Endre Károly, KristófRamalho-Santos, JoaoEndres, KristinaRamaraj, PanduranganEngelberg, DavidRamírez, CarmenEngelhardt, Paul E.Ramirez-Peña, EsmeraldaEngevik, MelindaRamos, DomingoEnns, CarolineRamos, SoniaErdélyi, MártaRampon, ChristineEri, RajaramanRandall-Demllo, SarronEsatbeyolgu, TubaRankin, GaryEscames, GermaineRanucci, DavidEscudero, OlgaRanzato, EliaEspadas Villanueva, IsabelRapeanu, GabrielaEspen, LucaRasola, AndreaEspino, JavierRattan, SureshEspinoza, Jorge LuisRaugei, GiovanniEsquifino, Ana IsabelRavanidis, StylianosEsteve-Romero, JosepRawel, HarshadraiEstrela, José M.Rayman, Margaret PEufemi, MargheritaRe, LambertoEuler, GerhildRebollo-Hernanz, MiguelEva, Martínez-PinillaReeve, VivienneEvans, CliveRegiec, AndrzejEvans, KervinRekawiecki, RobertExbrayat, Jean-MarieRembiałkowska, EwaEzhov, MaratRen, XiaoyuanFabiani, RobertoRenò, FilippoFadda, AngelaRenshaw, DerekFaitot, FrancoisRescigno, AntonioFalcão, Soraia I.Restivo, Francesco M.Falnoga, IngridRestuccia, DonatellaFalqué López, ElenaRetta, Saverio FrancescoFang, JunnanRety, StephaneFang, SungSoonRey, María-DoloresFanizzi, Francesco PaoloRhee, Jin-KyuFanti, LauraRibaudo, GiovanniFarabegoli, FulviaRibeiro, ArturFarè, SilviaRibeiro, DanielaFato, RomanaRibeiro-Barros, Ana I.Favati, FabioRicci, AntonellaFavre-Réguillon, AlainRicci, AriannaFeás, XesúsRiccò, MatteoFedin, MatveyRichards, Dylan J.Fedorova, MariaRichter, SusanFedulov, Alexey V.Rieg, TimoFekete, AgnesRiemer, JanFelgueiras, HelenaRijo, PatríciaFeng, Chia-HsienRimbach, GeraldFerens-Sieczkowska, MirosławaRinaldi, CarmenFeresin, Rafaela G.Rinaldi, TeresaFerlazzo, NadiaRingseis, RobertFernandes, Ana S.Rinnerthaler, MarkFernandes, JolynRios-Covian, DavidFernández Barbero, GerardoRiuzzi, FrancescaFernandez, Bernadette O.Rivas, FatimaFernández, José A.Rivera, Víctor M.Fernandez, MercedesRizvi, Syed A. A.Fernández-García, M. IsabelRizzarelli, EnricoFernández-Pérez, LeandroRizzi, LauraFerraboschi, PatriziaRizzo, GianlucaFerrante, ClaudioRizzolo, AnnaFerrarese, CarloRoca, JordiFerrari, Silvia MartinaRocchetti, GabrieleFerreira, Paula M.Rochani, AnkitFerreiro-González, MartaRoche, EnriqueFerrer, BeatrizRochfort, KeithFerro, DomenicoRodrigo, Sara MoralesFerro, RiccardoRodrigues, FranciscaFerroni, LorenzoRodrigues, JoãoFeveile Young, JetteRodrígues-Díez, RaquelFidalgo, AlexandraRodrigues-Diez, RaulFifere, AdrianRodríguez Bernaldo De Quirós, AnaFilep, János G.Rodríguez, SorayaFilgueira, CarlyRodríguez, Víctor ManuelFilip, AdrianaRodriguez-Lopez, Antonio D.Filip, LorenaRodriguez-Miguelez, PaulaFilip, PetrRodríguez-Solana, RaquelFilipek, AnnaRodríguez-Yoldi, María JesúsFilipovic, Milos R.Rogalska, JustynaFillmore, PaulRogobete, Alexandru FlorinFini, Mehdi ARoh, MiinFinley, John WeldonRoh, SanghoFinno, CarrieRohn, SaschaFiorillo, ClaudiaRoje, DamirFirczuk, MalgorzataRokavec, MatjazFiset, SylvainRolinec, MichalFisher, Aron B.Romanelli, Maria GraziaFiszer, AgnieszkaRomani, AndreaFlieger, JolantaRomani, PatriziaFlorek, EwaRomeo, RobertoFlorencio, Francisco J.Romero, AlejandroFlores-Bellver, MiguelRomero, Francisco JavierFlorescu, MonicaRoncarati, AlessandraFogacci, FedericaRondeau, PhilippeFogagnolo, PaoloRoosterman, DirkFontanay, StéphaneRosa, AntonellaFontanesi, FlaviaRosado, CatarinaFontanini, DeboraRosca, Ana MariaFonte, PedroRosi, MarzioFontés, MichelRosic, NedeljkaForero-Quintero, Linda S.Ross, IanForesti, RobertaRos-Santaella, Jose LuisForma, EwaRossi, ClaudioFormanowicz, DorotaRossi, GianpaoloFormenti, DamianoRossi, Jean-ChristopheFornai, FrancescoRossi, MiriamForte, ElenaRosso, ChiaraForti, LucaRostoker, GuyFouda, AbdelrahmanRoth, MichaelFox, JulieRothbauer, MarioFraga, Cesar G.Rouault-Pierre, KevinFraile, LorenzoRouphael, YoussefFrançois, CasasRozalski, MarcinFrank, SašaRua, DiegoFrankenberg-Dinkel, NicoleRudnitskaya, EkaterinaFratini, FilippoRudyk, OlenaFredotović, ŽeljanaRudzińska, MagdalenaFreire, M. SoniaRuf, Viktoria C.Frezza, ClaudioRui, LixinFrolov, AndrejRuiz Laza, RocíoFrutos-Fernández, María JoséRuiz Téllez, TrinidadFrydrychova, Radmila CapkovaRuiz, MarioFthenakis, GeorgeRumbos, ChristosFujii, YoshiharuRummey, ChristianFujimori, KoRusso, DanielaFujita, MasayukiRusso, MarinaFujiwara, KeigiRusso, Mario VincenzoFukudome, AkihitoRustad, TuridFulignati, SaraRuthstein, SharonFulle, StefaniaRutigliano, MariacinziaFurdui, Cristina MariaRuzik, LenaFürnsinn, ClemensRybczyńska-Tkaczyk, KamilaFusco, RobertaRybtsov, StanislavFuso, AndreaRymaszewska, JoannaFuu-Jen, TsaiRyu, ChangseonGabandé-Rodríguez, EnriqueRyu, DongryeolGabriel, RobertRywaniak, JoannaGac, PawełRzemieniec, JoannaGafencu, AncaRzepecka-Stojko, AnnaGagaoua, MohammedRzewuska, MagdalenaGago-Fuentes, RaquelSaastamoinen, MarkkuGajewska, A.Sabatier, Jean-MarcGajger, Ivana TlakSabatino, LauraGalanakis, Charis M.Saccà, Sergio ClaudioGalaris, DimitriosSachadyn-Król, MonikaGalatou, EleftheriaSadowska-Krępa, EwaGalbiati, MariaritaSadowski, MartinGalea, IanSaez, CarmenGalic, AnteSafirstein, RobertGalindo-Villegas, JorgeSahrawy, MariamGaliniak, SabinaSahu, Ravi PGall Troselj, KoraljkaSaidu, Nathaniel Edward BennettGáll, ZsoltSainz, Rosa MGalli, FrancescoSaito, SatoshiGallo, MonicaSaito, YoshinobuGalluccio, MicheleSaito, YoshiroGallyas, FerencSak, Jarosław J.Galvez, JorgeSakakibara, HiroyukiGálvez, JulioSala, GessicaGaman, Mihnea-AlexandruSalas, José BlancoGamberi, ChiaraSalas-Huetos, AlbertGambini, JuanSaldanha, SabitaGamon, Luke FrancisSalemi, MicheleGanau, MarioSalhab, HassanGanesan, KumarSalifoglou, AthanasiosGantner, MagdalenaSalinska, ElzbietaGaravaglia, Maria LisaSalmon, AdamGarbayo, InésSalto-Gonzalez, RafaelGarcía Gonzalo, FrancescSalucci, SaraGarcía, ConcepciónSalvo, AndreaGarcía, GabrielaŠamec, DunjaGarcía, José JoaquínSamhan-Arias, AlejandroGarcia, SamuelSamuels, IvyGarcía-García, Rosa MaríaSanak, MarekGarcia-Medina, José JavierSánchez, Jesus LozanoGarcía-Otín, Ángel LuisSánchez-Bueno, FranciscoGarcia-Santamarina, SarelaSanchez-Quesada, José LuisGard, PaulSancho, M. TeresaGareth, NyeSandbichler, AdolfGarrido-Fernández, AntónioSanfeliu, CoralGarten, RyanSanjeewa, Kalu Kapuge AsankaGarweg, Justus G.Sansbury, Brian E.Gasparrini, MassimilianoSansone, ClementinaGates, ColinSantabarbara, J.Gautheron, JérémieSantacroce, LuigiGawel, KingaSantanam, NaliniGaweł-Bęben, KatarzynaSanti, ClaudioGawlik-Dziki, UrszulaSantini, AntonelloGazerani, ParisaSantoro, MassimoGebicki, JanSantos De Almeida, TâniaGee, Heon YungSantos, Ana CláudiaGęgotek, AgnieszkaSantos, Andreia A.F.Genovese, ClaudiaSantos, JerranGentile, CarlaSantos, RenatoGentile, FabrizioSantulli, GaetanoGeny, BernardSanz, AnaGeorgakilas, AlexandrosSara, FranceschelliGeorgiev, Georgi As.Sarecka-Hujar, BeataGerardi, CarmelaSaretzki, GabrieleGerasimenko, OlegSarsour, EhabGerding, HeinrichSartore, GiovanniGericke, AdrianSasaki, YoGerm, MatejaSassoli, ChiaraGerothanassis, Ioannis P.Satake, MasayukiGhadiri, MalihehSatish, LakkakulaGhag, GauravSato, TakashiGhiasi, Seyed MojtabaSatoh, MasahikoGhidini, MicheleSatou, TadaakiGiacomello, EmilianaSattler, WolfgangGiallauria, FrancescoSattley, W. MatthewGiannenas, IliasSaurina, JavierGianotti, AndreaSavic, Ivan M.Gieseg, Steven P.Savion, NaphtaliGil, MinchanSawada, KojiGiles, GregorySawada, ShojiroGilloteaux, JacquesSawicki, TomaszGil-Martín, EmilioSazonova, Margarita A.Gil-Muñoz, RocioScala, ValeriaGiménez Bastida, Juan AntonioScalabrin, ElisaGiordano, AntonioScalcon, ValeriaGiordano, AnttonioScarafoni, AlessioGiordano, ElenaScarlata, EleonoraGiordano, MariaSchaefer, Michael K. E.Giordano, SamanthaSchalinskie, KevinGiotta, LiviaSchalkwijk, CasperGiovanna, Di EmidioSchechter, Alan NeilGiovannelli, LisaSchiavone, AchilleGiovannelli, PiaSchiavone, MarcoGiovannoni, RobertoSchiavone, StefaniaGiovinazzo, GiovannaSchiller, JürgenGiri, HemantSchilling, JoelGirolami, FlaviaSchinnerl, JohannGiron-Gonzalez, Maria D.Schipper, Hyman M.Gismondi, AngeloSchleicher, Erwin D.Giudetti, AnnaSchlicker, EberhardGiuffrè, Angelo MariaSchlüter, Klaus-DieterGiuffrida, DanieleSchmidt, EdGiulianini, Piero GiulioSchmitz, MatthiasGiuliano, MichelaScholkmann, FelixGiunta, SalvatoreSchomburg, LutzGiurisato, EmanueleSchöttker, BenGiuseppina, BasiniSchuchardt, MirjamGiustarini, DanielaSchulze, JohannesGiusti, Anna MariaSchunke, Kathryn JaquesGiusti, LauraSchwarzinger, BettinaGłąbska, DominikaŚcibisz, IwonaGladysheva, Inna P.Scicchitano, PietroGlibowski, PawełScimone, ConcettaGligor, FeliciaScognamiglio, MonicaGniazdowska-Piekarska, AgnieszkaScorziello, AntonellaGodfrey, RineshSeca, Ana M. LGoffart, SteffiSechi, Leonardo A.Gohar, EmanSecondo, AgneseGoldman, Irwin L.See Hoe, Louise E.Gołębiowski, TomaszSeeds, Michael CGolinelli, Marie-PierreSegatto, MarcoGoljanek-Whysall, KasiaSegura, InmaculadaGomaraschi, MonicaSeiquer, IsabelGómez Orte, Eva MaríaSekara, AgnieszkaGomez, JesusSellars, JonathanGómez-Torres, Maria JoséSELLES, BenjaminGonçalves, SandraSeluanov, AndreiGonkowski, SlawomirSendra, EstherGonzalez, AntonioSenoner, ThomasGonzález, JavierSenz Diez, María TeresaGonzalez, Michael JSeo, DaisukeGonzález, SoniaSeo, Young-KwonGonzález-Álvarez, JuliaSerefko, AnnaGonzalez-Bulnes, AntonioSereikaite, JolantaGonzalez-Casado, AntonioSergi, ConsolatoGonzález-García, JorgeSerio, FrancescoGonzález-Iglesias, HéctorSerreli, GabrieleGonzález-Manzano, SusanaSetyaningsih, WidiastutiGonzález-Minero, Francisco JoséSeverino, PaoloGonzález-Pérez, José A.Seydel, KarlGonzález-Redondo, PedroSgherri, CristinaGoodwani, SunilShackelford, Rodney E.Goraj, WeronikaShakeri, MajidGorgoulis, VassilisSharma, IshaGori, AntonellaSharman, Edward H.Gornas, PawelShcherbik, Natalia V.Gorniak, PatrykSherlock, Laurie G.Górnicka, MagdalenaSherman, PhilipGorynski, KrzysztofShertzer, Howard G.Goswami, Prabhat C.Shibata, YasushiGoto, TakaharuShieh, Tzong-MingGrabarczyk, MałgorzataShienfeld, YehudaGraber, TysonShih, Jing-WenGraczyk-Jarzynka, AgnieszkaShiina, MarisaGradalski, TomaszShikama, YosukeGradinaru, RobertShikov, AlexanderGramignoli, RobertoShim, Joon W.Granados-Principal, Sergio M.Shimanouchi, ToshinoriGranica, SebastianShimosawa, TatsuoGrant, GeorgeShin, YoungjaeGrasa, LauraShmarakov, IgorGrasso, GiuseppeShukitt-Hale, BarbaraGraziella, MessinaShukla, KirtikarGreenlief, C. MichaelShults, Nataliia V.Greetham, DarrenSiatka, TomášGregić, MajaSiatkowski, IdziGregory, Stephen L.Šic Žlabur, JanaGrider, ArthurSicari, VincenzoGrigoraš, Anca GiorgianaSidoti, AntoninaGrigoriev, I. P.Sieber, FritzGrigsby, Christopher L.Sieniawska, ElwiraGrilli, AlfredoSignorile, AnnaGrimm, Marcus O. W.Sigusch, Bernd W.Grimm, Wolf-DieterSikalidis, AngelosGrossi, GiancarloSikazwe, DonaldGrosso, GiuseppeSilaghi-Dumitrescu, RaduGruhlke, Martin C. H.Silswal, NeerupmaGrumati, PaoloSilva Moura, Elisabete FerreiraGruzman, Arie-LevSilva, Amelia MGrzegorczyk, IzabelaSilva, BrancaGrzelak-Błaszczyk, KatarzynaSilva, João PedroGrzesiak, JakubSilva, PaulaGuerriero, GiuliaSilva, PedroGuescini, MicheleSilvagno, FrancescaGugliandolo, EnricoSilvestre, Armando J. D.Guillén-Bejarano, RafaelSilvestre, SamuelGuiné, RaquelSilvestri, BrigidaGujski, MariuszSilvestri, CristianGulic, TamaraSimal, JesusGulisano, MassimoSimeonov, VasilGunn, TeresaSimes, DinaGuo, BinSimitzis, PanagiotisGuo, Chih-HungSimm, AndreasGuo, FenghaiSimó, RafaelGursoy, UlviŠimoník, OndřejGutierrez-Merino, CarlosSimonini, SaraGutsaeva, DianaSimón-Vázquez, RosanaGuyonnet, BenoîtSinanoglou, VassiliaGuzmán, José MiguelSingh, AbhishekGvozdjaková, AnnaSingh, AnilHa, EunyoungSINGH, JASHBIRHaanes, KristianSingh, PradeepHabas, KhaledSiniscalco, DarioHafer, CarstenSiracusa, RosalbaHailfinger, StephanSiraki, Arno G.Hajieva, ParvanaSjoholm, AkeHalagarda, MichałSkała, EwaHaller, Steven T.Skendi, AdrianaHallmann, EwelinaSkirtach, AndreHamaguchi, MasahideSkoko, John J.Hamano, TadanoriSkouta, RachidHamilton, ChrisSkov, VibeHamilton, ShannaSkovsted, Gry FrejaHammond, Billy R.Skroza, DanijelaHampton, Mark B.Skrypnik, Liubov N.Hanaka, AgnieszkaSkurikhin, Evgenii GermanovichHanania, MichelSlade, DeaHancock, JohnSlivka, Dustin RusselHankinson, OliverSliwinska, AgnieszkaHano, ChristopheSliwinski, TomaszHansen, Jason M.Sliwka, Hans-RichardHansíková, HanaSłomińska-Wojewódzka, MonikaHanus-Fajerska, EwaSlominski, AndrzejHardtke-Wolenski, MatthiasŚlusarczyk, SylwesterHargreaves, IainSmeriglio, AntonellaHarrison, SabineSmolen, SylwesterHartman, MariuszSmolková, KatarinaHarvey, AlexandraSmuder, Ashley J.Hasan, RaquibulSneddon, JenniferHasegawa, MorifumiSnowden, TimothyHasenstein, Karl HSobeh, MansourHashimoto, YoshitakaSobiech, PrzemysławHasse, SybilleSobierajska, KatarzynaHatae, NoriyukiSobocanec, CSandraHattinger, Claudia M.Sobota, Radoslaw M.Haybaeck, JohannesSocha, RobertHazai, LászlóSolano, FranciscoHe, QuanSoler Valls, Ana JosefaHecel-Czaplicka, AleksandraSoleti, RaffaellaHegyi, EszterSolingapuram Sai, Kiran KumarHeisinger, StephanSomlyai, GáborHeitmar, RebekkaSone, HidekoHeller, JanoschSong, Dae KyuHenkel, JaninSong, HailongHenry, Ryan A.Sonntag, KaiHer, Lu-ShiunSorg, OlivierHerbet, MariolaSoriano, José MiguelHermesz, EditSoriano-Arroquia, AnaHernan, AmandaSoriente, AlessandraHernández, ÁngelSorokin, VitalyHernández, FranciscaSorrell, J. MichaelHerráez-Hernández, RosaSORRENTINO, RAFFAELLAHerrero, EnriqueSosa Díaz, TeresaHerrero, María JesúsSousa, Maria JoãoHershfinkel, MichalSousa, MárioHervás-Marin, DavidSoveral, GraçaHeydemann, AhlkeSowa, IreneuszHeyse, AlexSpallarossa, AndreaHiggins, Paul J.Spampinato, Simona FedericaHildreth, SherrySpassov, SashkoHilhorst, MarcSpiers, JeremeHinds, Terry D.Spinelli, FrancescoHirata, YokoSpingler, BernhardHirota, KiichiSpínola, VítorHirotaka, KanedaSpizzirri, U GianfrancoHoffman, JessicaSpletter, MariaHoffmann, Michael MarcusSquillacioti, CaterinaHojs, RadovanŠrámek, JanHollenbach, MarcusSrečec, SinišaHolt, RobertaSrinivasan, RahulHoma, SherylSrivastava, SarikaHomma, TakujiroSrivatsan, MalathiHong, Jiann-RueySroka, ZbigniewHöög, Jan-OlovStacewicz-Sapuntzakis, MariaHorstkorte, RüdigerStadnyk, AndrewHortigón-Vinagre, MaríaStagos, DimitriosHortobágyi, TiborStahl, WilhelmHorvat, DanielaStanek, AgataHorváth, Eszter MáriaStanila, AndreeaHorváth, GabriellaStankova, IvankaHošek, JanStanley, Jone A.Hosohata, KeikoStanley, RogerHoung, Jer-YiingStarčević, AntonioHritcu, LucianStarowicz, MałgorzataHsia, Shih-MinStarzak, KarolinaHsiao, GeorgeStefanelli, ManuelaHsieh, Chang-WeiStefania, FilosaHsieh, Ming-JuStefano, Antonio DiHsieh, Wen-TsongStefanon, BrunoHsin, Kun-YiStefanowicz-Hajduk, JustynaHsu, Tsai-ChingStefanucci, AzzurraHuang, Chun-YungSteinbrenner, HolgerHuang, Guan-JhongSteiner, Joseph P.Huang, Huey-ChunStellavato, AntoniettaHuang, Hui-ChiStepanic, VisnjaHuang, Shih-YiSteullet, PascalHuber, OtmarStewart, AdeleHubicka, UrszulaStilli, DonatellaHuchzermeyer, BernhardStipa, PierluigiHuertas, Jesús R.Stiuso, PaolaHung, Shao-WenStixova, LenkaHung, Shih-YaStobdan, TseringHuppé-Gourgues, FrédéricStockmann, ChristianHura, TomaszStoian, Anca Mihaela PanteaHusvéth, FerencStojko, JerzyHwang, In KooStoleru, VasileHybertson, Brooks M.Stone, William L.Hytti, MariaStorchi, PaoloIanora, AdriannaStornaiuolo, MarianoIapichino, GiovanniStorsberg, JoachimIbrahim, SalamStoyanova, AlbenaIchii, HirohitoŠtrac, Dubravka ŠvobIchikawa, HiroshiStrappe, PadraigIentile, RiccardoStrati, Irini F.Igual, MartaStratmann, BerndIida, KaorukoStroffekova, KatarinaIlmarinen, TanjaStrube, JochenIm, Seung-SoonStrungaru, Stefan-AdrianImpellizzeri, DanielaStrychalski, JanuszImperatore, ConcettaStrzemski, MaciejInagaki, HidetoshiStuart, JeffreyInfascelli, FedericoStubbs, EvanInnchi, LeeStushnoff, CecilInoue, AtsukoStutz, BernardoInoue, HirofumiSu, HuaInoue, KazukiŠubarić, DragoIoannou, IrinaSubramani Paranthaman, BalasubramaniIonescu, Elena RodicaSud’ina, Galina F.Ionut, Ioana A.Suen, Jau-LingIrakli, MariaSugawara, AkihiroIrato, PaolaSuh, Hyung JooIrena, NalepaSuharoschi, RamonaIsemura, MamoruSujak, AgnieszkaIshikawa, KiyotakeSukseree, SupawadeeIshitsuka, YosukeSuliburska, JoannaIslam, Mohammad MirazulSulová, ZdenkaIsola, GaetanoSuomela, Jukka-PekkaItabe, HiroyukiSurdacki, AndrzejIto, FumiakiSureda, AntoniItoh, KenSurguchov, AndreiItoh, ToshimasaSuzuki, KatsuhikoIuliano, RodolfoSuzuki, NorioIvanova, ElenaSuzuki, ShugoIves, StephenSuzuki, TakafumiIwai, AtsushiSuzuki, ToshikazuIwaoka, MichioSvobodová, JanaIyer, ShilpaSvoronos, Paris D. N.Izdebska, MagdalenaŚwiątek, PiotrJaaro-Peled, HannaŚwiątkiewicz, MałgorzataJabłońska-Trypuć, AgataŚwieca, MichałJaburek, MartinSykłowska-Baranek, KatarzynaJacenik, DamianSymonds, Michael E.Jacevic, VesnaSytar, OksanaJackson, William F.Sytykiewicz, HubertJadavji, Nafisa M.Szakiel, AnnaJaganjac, MoranaSzaleniec, MaciejJaime, Diego FrancoSzászi, KatalinJakobusic Brala, CvijetaSzatmari, Erzsebet MariaJakubczyk, EwaSzepesi, AgnesJakubowski, HieronimSzerszunowicz, IwonaJala, Venkatakrishna RaoSzewczyk, AgnieszkaJamka, MałgorzataSzewczyk, KatarzynaJamróz, EwelinaSzkaradkiewicz, AndrzejJamwal, RohitashSznarkowska, AlicjaJanda, ElzbietaSzopa, AgnieszkaJanda, KatarzynaSzóstek-Mioduchowska, AnnaJang, Byoung KukSztanke, MałgorzataJaniszewska-Turak, EmiliaSzulc-Dąbrowska, LidiaJankauskienė, JulėSzumny, AntoniJanowiak, Blythe E.Szutowicz, AndrzejJantzie, Lauren L.Szwajca, AnnaJara Palacios, María JoséSzymandera-Buszka, KrystynaJarmuszkiewicz, WiesławaSzymanski, JedrzejJarocka-Karpowicz, IwonaTabuchi, MasashiJarzebski, MaciejTachibana, YuyaJastrzebska, BeataTada, KoheiJaworowska, AgnieszkaTae-Jin, KimJeandet, PhilippeTafuri, SimonaJedrejek, DariuszTähtinen, PetriJembrek, Maja JazvinšćakTai, Dar-FuJena, Prasant KumarTaiar, RedhaJeon, SookyoungTaira, JunseiJerala, Nataša KopitarTajiri, NaokiJerebtsova, MarinaTakahashi, HidenoriJerkowić, IgorTakahashi, HideyukiJeschke, UdoTakahashi, KenJeung, Eui-BaeTakano, KatsuraJežek, PetrTakasawa, ShinJiang, Lin-HuaTakatani-Nakase, TomokaJobe, TimothyTakaya, TomohideJoers, ValerieTakayama, FusakoJohnston, CarolTakegahara, NorikoJones, MerielTakemori, HiroshiJong, ChianjuTakemura, MihoJoo, Nam-SeokTamba, Bogdan IonelJorge, FeitoTambuwala, MurtazaJosé Tauler Riera, PedroTanabe, KatsuyukiJoshi, Pushpa RajTanaka, AtsushiJuhász, TamásTanaka, MasaruJukić Špika, MajaTanase, CorneliuJumbo-Lucioni, PatriciaTang, Bor LuenJung, Young SungTang, Soon YewJung, YoungSukTanini, DamianoJunichi, KitanakaTanito, MasakiJurkowska, HalinaTantos, ÁgnesJuszczak, LesławTaoka, YousukeJyotsna, MishraTappia, ParamjitKabil, OmerTarozzi, AndreaKabir, Mohammad FaujulTARRAGO, LionelKaczmarek, BeataTasso, BrunoKadowaki, DaisukeTatullo, MarcoKaether, ChristophTava, AldoKafarski, PawelTavazzi, BarbaraKafarski, PawełTedesco, IdoloKageyama, HakutoTeiber, JohnKaja, SimonTejero, JesúsKajihara, MikioTelegina, Darya V.Kalayda, GannaTena, NoeliaKale, AbhijitTeng, Ba-BieKalinin, VladimirTenore, GianKaltsatou, AntoniaTeodoro, JoãoKam, AntonyTeragawa, HirokiKameshima, SatoshiTermini, JohnKamieniczna, MarzenaTerrasson, VincentKamiński, PiotrTerzaghi, William BryanKamolz, Lars-PeterThackray, AlanaKamzolova, SvetlanaThangavel, ChellappagounderKanai, MasashiThomas, BijuKanamori, TakaoThomas, JohnKanda, NaokoThounaojam, Menaka ChanuKandylis, PanagiotisThummuri, DineshKang, DongchulTian, Shung WuKang, JunsuTiano, LucaKang, Kyung PyoTiboni, Gian MarioKaňková, KateřinaTilley, DouglasKannan, RamTiperciuc, BrindusaKant, SashiTirillini, BrunoKanugula, Anantha KoteswararaoTitze, JeanKao, Chai-LinToapanta, Franklin R.Kappus, RebeccaTodorova, DessislavaKapur, NeerajToepel, JörgKapusta, IreneuszTofalo, RosannaKar, AdwitiyaToh, Ban-HockKarcz, DariuszTomaszewska, EwaKardassis, DimitrisTonetti, LorenzoKargl, JuliaTong, JunchaoKarikas, George-AlbertTonissen, Kathryn FKarner, CourtneyTonni, GabrieleKarwowska, MałgorzataTonolo, GiancarloKase, SatoruTorre, Maria LuisaKashfi, KhosrowTorres Fuentes, CristinaKashihara, NaokiTorres Perez, María DoloresKashiwakura, IkuoTorres-Cuevas, IsabelKashtalap, VasilyTosti, ElisabettaKaškonienė, VilmaToth, PeterKataoka, HiroyukiTovar, JuscelinoKato, MasayaTower, JohnKato, TakamitsuTrabace, LuigiaKato, Takamitsu ATrabalzini, LorenzaKato, YasumasaTraina, GiovannaKatsoris, PanagiotisTramonti, AngelaKauppinen, AnuTravers, Jeffrey BryantKaur, GurvinderTrcek, JanjaKawakami, SusumuTrček, JanjaKawakita, HidetakaTrentini, AlessandroKawamura, TakujiTrevisan, AndreaKawanami, DaijiTribulova, NarcisKawata, KazumiTroedsson, Mats H.T.Kawiak, AnnaTrofimov, Aleksei V.Kayano, Shin-ichiTROMBETTA, MARIAKazimierczak, RenataTroppmair, JakobKeinänen, MarkkuTrucillo, PaoloKejík, ZdeněkTrusca, Georgeta VioletaKelleher, Shannon L.Trzcińska, MonikaKeller, Amy CelesteTsai, JamesKello, MartinTsai, Jen-ChiehKensler, Thomas W.Tsai, Kun-LinKhan, AmjadTsai, Pei-ShiueKhan, MohsinTsai, Pi-JenKhan, NababTsantarliotou, Maria PKhanna, HemantTsantili-Kakoulidou, AnnaKhlebnikov, Andrei I.Tscheliessing, RupertKicel, AgnieszkaTschernig, ThomasKicińska, AnnaTseng, Chih-HuaKieliszek, MarekTsopmo, ApollinaireKikowska, MałgorzataTsoukalas, DimitrisKikuchi, HidehikoTsubota, AkihitoKikusato, MotoiTsuchiya, MasahikoKikuzaki, HiroeTufarelli, VincenzoKilanowicz, AnnaTundis, RosaKilari, SreenivasuluTurano, PaolaKim, BongleeTurrini, FedericaKim, DongukTuttolomondo, AntoninoKim, DoyeonTuuminen, TamaraKim, Eun-HeeTyan, Yu-ChangKim, Gi JinTzen, Jason T.C.Kim, Hee KeeTzeng, Chii-ReuyKim, Ho-ShikTzeng, Shiang-JongKim, Hyeung-RakUberti, DanielaKim, Hyung-SikUccelletti, DanielaKim, JongminUchida, TakeshiKim, Ki HyunUfnal, MarcinKim, KijinUllah, Md AshikKim, Kil-SooUmehara, MikihisaKim, Mi-hyunUrban, MilanKim, Min-HyunUrbanelli, LorenaKim, Min-SooUrbaniak, AlicjaKim, Nam DeukUrbanucci, AlfonsoKim, SeonghunUrra, José MiguelKim, Seung-HyunUrsoniu, SorinKim, Sun YeouUsuki, ToyonobuKIM, Sung JoonUwai, KojiKim, Sung-HoonVacante, MarcoKim, Yong-IckVáclavíková, RadkaKim, Young-MoVafiadaki, ElizabethKimura, Ken-ichiVaiman, DanielKind, Karen L.Vairetti, MariapiaKinder, DavidVaisman, BorisKishikawa, NaoyaValasi, IreneKishimoto, DaigaVale, PauloKiss, BélaValente, Nicola AlbertoKiss, RitaValenti, MariaKlavins, MarisValentine, Rudy J.Kleeff, JörgValentová, KateřinaKleier, Daniel A.Valenzuela, Juan LuisKlein, David C.Valles, Soraya L.Klein, ErikValletta, AlessioKleinridders, AndréVamanu, EmanuelKleinstein, Sarah E.Van Der Laarse, Willem J.Kleist, Thomas J.Van Dyke, MichaelKlotz, JamesVan Gisbergen, Marike W.Kluczyk, AlicjaVan Saen, DorienKmiecik, DominikVányolós, AttilaKnez, DamijanVáradi, JuditKnezevic, JelenaVarikuti, SanjayKo, GladysVarillas, DavidKO, Kam MingVasicek, OndrejKobayashi, SatoruVasileiadis, IoannisKobeissy, FirasVasin, Mikhail V.Kobus-Cisowska, JoannaVassallo, AntonioKoch, WojciechVassallo, NevilleKoike, HarukiVaudry, DavidKojima, HajimeVavvas, Demetrios G.Kojima-Yuasa, AkikoVaz, Josiana A.Kokkola, TarjaVazquez, GuillermaKokoska, LadislavVeatch-Blohm, MEKokoszyński, DariuszVega Alvarez, Jose AntonioKolasa-Wołosiuk, AgnieszkaVega, Jose MariaKolesar, Jill MarieVejux, AnneKolesińka, BeataVelena, AstridaKolodziejczyk, JoannaVelentzas, Athanassios D.Kolosova, NataliyaVelkov, ZhivkoKomada, MunekazuVellecco, ValentinaKomba, ShiroVenckunas, TomasKomis, GeorgeVenskutonis, Petras RimantasKomoike, YutaVeranic, PeterKomsta, ŁukaszVerardo, VitoKonieczka, PawełVergara, DanieleKonopelski, PiotrVergara, M. NataliaKonopka, AdamVerma, Anil K.Konopka, IwonaVernetti, Lawrence A.Kontek, BogdanVerni, MichelaKontogiorgis, ChristosVerri, TizianoKontos, Christos K.Versnel, HuibKopacz, AleksandraVestergaard, Mun’delanji C.Kopjar, MirelaVetrano, Ignazio GaspareKoprowski, PiotrVetvicka, VaclavKorać, PetraVezzani, BiancaKoryak, Yuri A.Viapiana, AgnieszkaKorybalska, KatarzynaVicario, NunzioKosmala, MonikaVictor, VictorKostecka-Gugala, AnnaVieira, Helena L. A.Kosters, AstridVignes, MichelKostomitsopoulos, Nikolaos G.Vignini, AriannaKostrzewa-Nowak, DorotaVijayakumar, SarathKostyn, KamilVikulina, AnnaKosuge, YasuhiroVilela, AliceKotani, KazuhikoVilla, ChiaraKouji, HaradaVillacampa, MercedesKoukounaras, AthanasiosVillalta, PeterKouretas, DimitriosVillani, Rehan MKoutelidakis, Antonios E.Viña, JoséKovac, StjepanaVinall, RuthKowalczewski, PrzemysławVinci, CristinaKowalczuk-Vasilev, EdytaVinci, Maria CristinaKowalska, JolantaVingolo, Enzo MariaKozakiewicz, MariuszVinson, JoeKozhevnikova, OyunaVirag, Jitka A.I.Koziorowska-Gilun, MagdalenaVirag, LaszloKozlov, Andrey V.Virgili, FabioKozłowska, MariolaVirto, MailoKrajka-Kuźniak, ViolettaVisconti, RobertaKrawczynski, KamilVisentin, MicheleKrebs, BerntVítámvás, PavelKrestinina, OlgaVitetta, LuisKrijt, JanVitiello, PeterKristian, TiborVitorino, Rui Miguel PinheiroKrokidis, MariosVitturi, DarioKról, EwelinaViuda-Martos, ManuelKrupa, RenataVivarelli, FabioKu, Kang-MoVlassopoulos, AntonisKuan, Yu-HsiangVoběrková, StanislavaKuban-Jankowska, AlicjaVodnar, Dan C.Kubatka, PeterVolcho, KonstantinKubica, PawełVolkov, VadimKubicova, LenkaVomhof-DeKrey, EmilieKubinová, RenataVon Ameln-Mayerhofer, AndreasKubinyi, MiklósVon Knethen, AndreasKues, Wilfried A.Von Lintig, JohannesKuhn, HartmutVukojevic, KatarinaKujawska, MałgorzataWada, JunKukula-Koch, WirginiaWada-Hiraike, OsamuKulbacka, JulitaWagner, AndreasKulczyński, BartoszWagner, AnikaKulminski, AlexanderWagner, Kay-DietrichKumar, AkhileshWahli, WalterKumar, VijayWan, LeiKumari, AshaWang, Chao-MinKumari, NeelamWang, Chin-KunKunz, WolframWang, Hui-minKuo, Cheng-ChinWang, JiongweiKuo, Ping-ChungWang, Mong-HengKuo, Yao-HaurWang, Shin-WeiKurasaki, MasaakiWang, Shu-HueiKurganov, BorisWang, Wen-DerKurihara, YukioWarzecha, ZygmuntKurpisz, MaciejWa̧sowicz, WojciechKursvietiene, LolitaWatanabe, MikikoKus, PiotrWeaver, JolantaKushkevych, IvanWebb, R. ClintonKuźnik, NikodemWegwitz, FlorianKwak, Jong HwanWei, Ming-ChiKwak, SeonyeongWeiss, SiegfriedKwakowsky, AndreaWeng, Ching FengKwiecien, JacekWenta, TomaszKwon, Ki-SunWest, James D.Kypreos, Kyriakos E.Weston-Green, KatrinaL. Pey, AngelWhite, RogerLa Rosa, PiergiorgioWhittaker, PeterLa Russa, DanieleWianowska, DorotaLa Spina, MartinaWidemann, EmilieLa Vignera, SandroWieczorek, EdytaLabbé, SimonWiernicki, IreneuszLabbozzetta, ManuelaWiesner-Reinhold, MelanieLabrou, NikolaosWietstock, PhilipLabudda, MateuszWilczyński, JacekLacaille-Dubois, Marie-alethWilde, JerzyLacham-Kaplan, OrlyWilhelm Stahl, WilhelmLachlan, MitchellWilkins, HeatherLachowicz, SabinaWilkinson, JennyLacina, LukasWilliams, RyanLadilov, YuryWillmore, William G.Lagalwar, SaritaWińska, KatarzynaLaganà, Antonio SimoneWithers, SarahLagouri, VasilikiWitting, Paul K.Lahart, IanWnuk, AgnieszkaLakk, MónikaWnuk, MaciejLama, AdrianoWójciak, KarolinaLaMarca, BabetteWójciak, MagdalenaLamichhane, SantoshWójcik-Pszczoła, KatarzynaLamponi, StefaniaWojdylo, AnetaLamy, ElsaWojnowski, WojciechLandi, MarcoWojtunik-Kulesza, Karolina AnnaLanducci, ElisaWojtyla, ŁukaszLangdon, SimonWoo, So-YounLangton, Abigail K.Wood, JeffLanner, Johanna T.Wood, KatherineLanza, GiuseppeWoźniak, GabrielaLapteva, MariaWoźniak, ŁukaszLarre, IsabelWrześniok, DorotaLarson, Richard AWrzosek, MałgorzataLasoń, WładysławWu, Chi-ReiLatek, DorotaWu, ErxiLatif, SajidWu, GongweiLatino, Maria ElenaWu, Shu-JuLatouche, CamilleWu, Wei-LiLattanzio, RossanoXu, MaonianLauretani, FulvioXuan, Tran DangLaurin, JérômeYadav, Pramod KumarLaursen, Kristian BruunYadav, VinitaLavandera, Jose LuisYager, DorneLavelli, VeraYagishita, YokoLavin, Martin F.Yakimova, LuidmilaLavoie, Jean-ClaudeYamada, SohsukeLawrenson, JohnYamaguchi, YusukeLax, PedroYamakoshi, KimiLazar, ZsofiaYamakuchi, MunekazuLe Clec’h, WinkaYamamoto, ToshiharuLe Guellec, DominiqueYamamoto, YusukeLe Romancer, MurielYamanaka, KeiichiLe, Anh VYamasaki, HideoLebaron, RichardYamashita, AtsushiLech, KrzysztofYamauchi, AkiraLechel, AndreYang, Chang-HaoLedgerwood, Elizabeth C.Yang, Chuen-MaoLedo, AnaYang, ChunzhangLee, Ching-KuoYang, GuangdongLee, Dae-SungYang, JunhuaLee, Eun-WooYang, Shun-FaLee, GihyunYang, Wei-hsiungLee, Hong-JinYano, AkiraLee, Hui JaiYao, JianLee, JihyunYasuda, KazukiLee, Jong-JerYee, Callista StephanieLee, JunsooYeh, Shu-LanLee, JunyeopYen, Feng-LinLee, Kyung-AhYen, Frances T.Lee, Sang-HanYeste, MarcLee, SanghoYim, Soon-HoLee, Shin-DaYin, JieLee, Sung KiYing, MingyaoLee, Tzong-ShyuanYochum, Gregory S.Lee, Tzu-TaiYoda, SatoshiLee, Wing-KeeYokoi, FumiakiLee, Yong SunYoo, Yeong-MinLefevre, Sophie D.Yoon, YisangLehotský, JánYoshida, Go J.Lei, WeiYoshino, HironoriLei, ZhentianYoshioka, NaokiLeicht, ChristofYou, Hye JinLeisz, SandraYou, SeungkwonLeiva-Brondo, MiguelYoussefian, LeilaLenaz, GiorgioYtrestøyl, TrineLendvai, GáborYu, Chang YeonLeng, TiandongYu, KitaokaLentini, GiovanniYu, Yung-LuenLeon, AntonioYuann, Jeu-Ming P.Leon, FranciscoYudina, LyubovLeón, JosefaYun, Hwi-YeolLeón, RafaelYurchenko, EkaterinaLeone, GemmaYushenova, IrinaLeoni, ValerioZabad, Rana K.Leontopoulos, StefanosZabetakis, IoannisLephart, Edwin D.Zacchia, MiriamLes, FranciscoZaguła, GrzegorzLescure, AlainZaid, HilalLesniak, WieslawaZaitsev, AlekseyLevart, AlenkaZakłos-Szyda, MalgorzataLever, Jeremie M.Zakrzewski, JerzyLevy, EmileZalewska, AnnaLewandowicz, GrazynaZałuski, DanielLewinska, AnnaZammit Mangion, MarionLi, Chia-JungZamyatnin, AndreyLi, GuohuiZanella, IsabellaLi, JineZanetic, MirellaLi, LianboZanón-Moreno, Vicente CalixtoLi, Po-HsienZapata Coll, Pedro JavierLi, ShiriZapico, Sara CasadoLi, WeiZapotoczny, SzczepanLi, XinboZaprutko, LucjuszLiagre, BertrandZarkovic, NevenLiao, Hung-JuZarnowski, TomaszLiao, Jyh-feiŻarowska, BarbaraLiao, Yung-FengZarrelli, ArmandoLiby, KarenZarzycki, Pawel K.Lim, Gareth E.Zawada, KatarzynaLim, Kyung-MinZawadzki, JarosławLim, Tae-GyuŻbikowska, AnnaLima, BeatrizZdarta, JakubLima, ElisabeteZecca, LuigiLimauro, DanilaZelina, PavolLin, Chang-ShenZella, DavideLin, Chih-LiZeng, XiangkangLin, Chun-PingZerbini, GianpaoloLin, Hai-ShuZerva, AnastasiaLin, Hsu YangZhan, YiqiangLin, Hui-HsuanZhang, CongqiangLin, Jer-AnZhao, WeiLin, Pei-HuiZhou, AiminLin, Shih-YiZiegler-Borowska, MartaLin, Tung-YiZimmer-Bensch, GeraldineLin, Wei-YongZingg, Jean-MarcLin, Yuh-YihZinnai, AngelaLindsay, AngusZiogas, VasileiosLio, DoZiolkowski, WieslawLiobikas, JuliusZitka, OndrejLionetti, VincenzoZitko, JanLionetto, Maria GiuliaZitz, UlrikeLiou, Ying-mingZiyatdinova, GuzelLipińska, BarbaraZłotek, UrszulaLitofsky, Norman ScottZoidis, EvangelosLitvinova, Ekaterina A.Zorov, Dmitry BLiu, Chia-ChiZotti, FrancescaLiu, Chung-JungZovko Končić, MarijanaLiu, Shing-HwaZucca, PaoloLiu, Yi-ChangZuccari, GuendalinaLiu, Yung-ChuanZugaza, José LuisLiu, ZijuanŻyżelewicz, Dorota

